# Airway emergency during general anesthesia in a child with plastic bronchitis following Fontan surgery: a case report

**DOI:** 10.1186/s40981-020-0311-5

**Published:** 2020-01-21

**Authors:** Hirofumi Nakamoto, Satoru Kayama, Mae Harada, Takahiro Honjo, Kinuko Kubota, Shigehito Sawamura

**Affiliations:** Department of Anesthesiology, Teikyo University School of Medicine, 2-11-1 Kaga, Itabashi-ku, Tokyo, 173-8605 Japan

**Keywords:** Plastic bronchitis, Fontan surgery, Lymphatic embolization, Airway emergency

## Abstract

**Background:**

Plastic bronchitis (PB) is a complication of Fontan surgery, results in the formation of mucus plug in the tracheobronchial tree, causing potentially fatal airway obstruction. We report critical airway emergency during general anesthesia in a child with plastic bronchitis.

**Case presentation:**

A 5-year-old boy was scheduled for intrapulmonary lymphatic embolization through percutaneous catheterization under general anesthesia. He underwent Fontan surgery at the age of 2 and frequently developed respiratory failure due to plastic bronchitis. After induction of general anesthesia and tracheal intubation, mechanical ventilation became difficult even with an inspiratory pressure ≥ 50 mmHg due to airway obstruction. He expectorated a large mucus plug through the tracheal tube after administration of sugammadex, naloxone, and flumazenil, and respiratory condition was stabilized thereafter.

**Conclusion:**

General anesthesia for a patient with plastic bronchitis should be planned with extracorporeal membrane oxygenation or cardiopulmonary bypass stand by.

## Background

Plastic bronchitis (PB) is a rare, but potentially fatal complication following Fontan surgery, a palliative procedure for patients with functional single ventricle. It is characterized by mucus plugs formed in the tracheobronchial tract and may cause life-threatening airway obstruction. Here, we present a case of difficult anesthesia management in a child with plastic bronchitis.

## Case presentation

A 5-year-old boy was scheduled for intrapulmonary lymphatic embolization through percutaneous catheterization. He underwent Fontan surgery at the age of 2 and frequently developed respiratory failure due to plastic bronchitis thereafter. His family history was unremarkable and the course of pregnancy was uneventful. Heart abnormalities were detected by fetal echocardiography. He was delivered transvaginally at 38 weeks 4 days with a weight of 2696 g. Diagnoses of a double outlet right ventricle, aortic valve atresia, aortic coarctation, and atrioventricular septal defect were made immediately after birth. Norwood surgery, Blalock-Taussig shunt, and Glenn surgery were performed 6 days, 1 year, 5 months, and 2 years after birth, respectively. Fenestrated Fontan surgery was performed 2 years 3 months after birth. Although surgery was completed uneventfully and Fontan circulation was successfully established, he developed dyspnea with decreased SpO_2_ 30 days after surgery. He discharged white-colored bronchial casts and a diagnosis of plastic bronchitis (PB) was made 44 days after surgery. His respiratory condition was refractory to conservative treatment such as low-fat diet, respiratory physiotherapy, as well as to medications including bronchodilators, expectorants, and pulmonary vasodilators, therefore, intrapulmonary lymphatic embolization through percutaneous catheterization was scheduled.

Preoperative findings were as follows: height, 115.2 cm (+ 1.0 SD); weight, 16.1 kg (− 1.0 SD); body temperature, 36.8 °C; heartbeat, 100 beats per minute; blood pressure, 98/50 mmHg; and SpO_2_, 87–89% (nasal oxygen 1 L/min). Jugular venous distention and a systolic murmur of grade I/IV on the Levine scale at the third intercostal space along the left sternal border were observed. Auscultation revealed normal respiratory sounds. The chest X-ray demonstrated cardiomegaly and pulmonary congestion. The electrocardiograph revealed a right bundle branch block and right ventricular hypertrophy. Echocardiography revealed fenestration and trivial common atrioventricular valve regurgitation without stenosis of IVC conduit. Chest CT was not performed because of the patient’s age. Resting SpO_2_ was 88–89% which decreased to 85% during exercise, accompanied by shortness of breath and cyanosis. Mucus plugs were discharged every 4–5 days.

On the morning of the surgery, a mucus plug was discharged; however, the patient had no fever and was active. To identify the site of lymphatic leakage by temporarily increasing lymph production, the patient ingested 3 peanuts 3 h before surgery. Upon entering the operating room, SpO_2_ was 73% under room air as the patient was crying. Standard monitoring and invasive arterial pressure measurement were started.

**Fig. 1:**
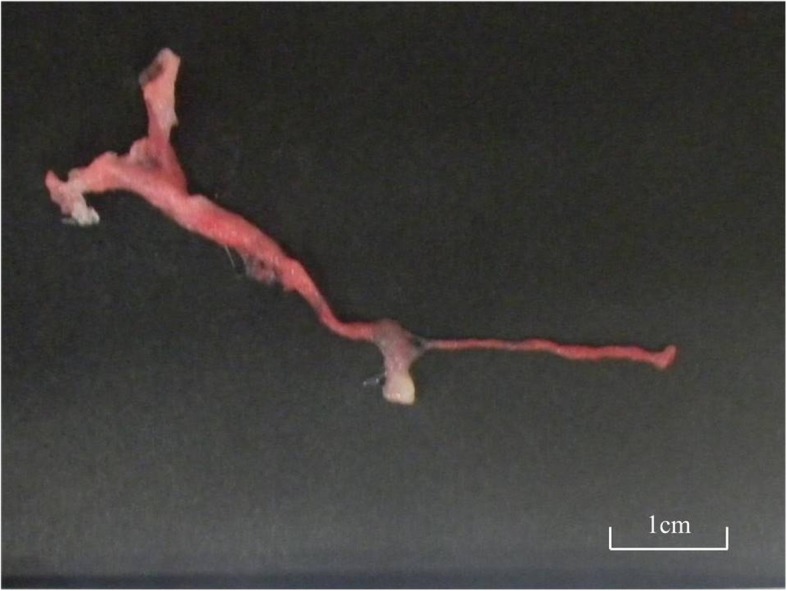
Mucus plug forming a branching bronchial cast discharged from the patient. Respiratory status improved significantly after its expectoration

After starting dopamine (3 μg/kg/min), anesthesia was induced using midazolam 1 mg, rocuronium 15 mg, fentanyl 30 μg, and sevoflurane 1.5%. During laryngeal deployment, a large amount of secretions was accumulated in the upper airway and mucosal swelling around the glottis was observed. After a 4.5-mm cuffed endotracheal tube was inserted, a slightly sticky, pale yellow, translucent mucus leaked out of the endotracheal tube, necessitating frequent suction. Five minutes after the intubation, expiratory CO_2_ became undetectable and mechanical ventilation became difficult, necessitating manual ventilation at a maximum inspiratory pressure of 50 mmHg or greater. Respiratory tract obstruction due to mucus plugs was suspected, and surgery was postponed. End-tidal CO_2_ (EtCO_2_) reached 120 mmHg, and SpO_2_ temporarily decreased to 60–70%, while mean blood pressure was maintained between 50 and 80 mmHg. Direct removal of the mucus plugs was attempted using a flexible bronchoscope, but it was impossible to observe the peripheral airways due to abundant mucus secretions. Moreover, the diameter of the endotracheal tube was too small, to allow manipulation of the bronchoscope, and the mucus plugs could not be adequately removed. Sugammadex 50 mg, naloxone 0.02 mg, and flumazenil 0.1 mg were administered to promote self-expectoration through cough reflex. Six million units of recombinant tissue-type plasminogen activator (rtPA) and 200 μg of salbutamol sulfate were administered intratracheally, and hydrocortisone sodium phosphate ester 50 mg was administered intravenously. Frequent intratracheal suction and postural drainage were performed. Subsequently, the cough reflex was triggered and a mucus plug in the form of a bronchial cast (approximately 5 cm in length) was discharged (Fig. 1) through the endotracheal tube. The patient resumed spontaneous breathing and his respiratory conditions stabilized after the expectoration.

As extracorporeal membrane oxygenation (ECMO) for young children was unavailable in our hospital, the patient was transferred to another institution for safety, where airway obstruction recurred and ECMO was introduced temporarily. Six days later, the patient was weaned from the ventilator and discharged with no signs of neurological sequelae.

## Discussion

Since its first report in 1971 [1], prognosis after Fontan surgery has improved through progress in surgical techniques and advances in perioperative management, with a 10-year post-operative survival rate of 95% [2]. However, various complications have been reported following surgery in the long term, such as heart failure, tachyarrhythmia, thromboembolism, pulmonary arteriovenous fistula, liver cirrhosis, protein-losing gastroenteropathy, and PB [3].

In PB, high central venous pressure inhibits lymphatic fluid drainage through the thoracic duct. As a result, the lymphatic fluid leaks into the bronchi and produces mucus plugs, which can form bronchial casts [4, 5]. These mucus plugs cause respiratory failure due to tracheobronchial tract obstruction, occasionally resulting in mortality. The incidence of PB is relatively rare (around 4%) [6]; however, its prognosis is poor, with a 5-year mortality of approximately 50% [7]. In the long term, therefore, PB is one of the most life-threatening complications of Fontan surgery.

Today, there are no established treatments for PB. Typically, treatments to improve cardiac function, fat-restricted diets, physical therapy, and pharmacotherapy are performed. In pharmacotherapy, expectorants, antibiotics, bronchodilators, steroids, heparin, and thrombolytics are used [8, 9]. When resistance to pharmacotherapy occurs, invasive treatments are performed, such as transcatheter intrapulmonary lymphatic embolization of intrapulmonary lymphatic vessels [10, 11], surgical thoracic duct ligation [12, 13], fenestration reopening [5, 14], and heart transplantation [15]. In our case, transcatheter intrapulmonary lymphatic embolization was planned because the patient had developed drug resistance, and the disease was worsening due to an increase in mucus plug discharge and repetition of pneumonia.

Scrupulous preanesthetic evaluation could possibly have helped to prevent the airway emergency in our patient. As the mucus plug had been discharged on the morning of surgery and a large amount of secretions and mucosal swelling were observed in the upper airway, the timing of operation should have been reconsidered. Moreover, preoperative steroids or thrombolytics might have been effective.

We selected general anesthesia with a cuffed endotracheal tube to achieve immobility intraoperatively and to secure the airway and respiration, as the procedures were planned through the internal jugular vein. A cuffed endotracheal tube was also appropriate as manual ventilation with a maximum airway pressure of 50 mmHg or higher was required during the respiratory tract obstruction caused by the mucus plugs. Although the bronchoscope worked poorly through the endotracheal tube (inner diameter 4.5 mm), we decided not to exchange the endotracheal tube intraoperatively because the risk to the patient was thought to be significant.

The ventilation mode during anesthesia in PB should be chosen carefully, as maintaining spontaneous breathing and cough reflexes may significantly aid in the expectoration of mucus plugs. We speculated that positive pressure ventilation pushed the mucus plugs deep into the peripheral airways and the elimination of cough reflexes made their expulsion difficult. Mucus plugs were expectorated after the recovery of the cough reflex, and the respiratory status was stabilized after the resumption of spontaneous breathing. Although spontaneous breathing is typically recommended to decrease pulmonary vascular resistance in Fontan circulation, there is also a risk of pulmonary vascular resistance elevation through the accumulation of CO_2_ in cases of insufficient spontaneous respiration.

In PB after Fontan surgery, we speculate that it is preferable to perform surgery in a facility where ECMO is immediately available. Our patient was transferred to a children’s hospital for the sake of safety, as ECMO was not available for young children in our institution. Respiratory tract obstruction recurred, and ECMO was initiated after the transfer. The use of ECMO in more invasive surgical treatments for PB has been reported [13, 14]. Given the high risk of critical airway obstruction, general anesthesia for a patient with PB should be planned with ECMO or CPB standby.

## Data Availability

Not applicable.

## Abbreviations

ECMO: Extracorporeal membrane oxygenation
EtCO_2_: End-tidal CO_2_
PB: Plastic bronchitis
rtPA: Recombinant tissue-type plasminogen activator
SpO_2_: Saturation of percutaneous oxygen
